# Retraction: Targeting a LncRNA P5848-ENO1 axis inhibits tumor growth in hepatocellular carcinoma

**DOI:** 10.1042/BSR-2018-0896_RET

**Published:** 2024-08-28

**Keywords:** ENO1, Hepatocellular carcinoma (HCC), LncRNA P5848, Therapeutic target

This Retraction follows an Expression of Concern relating to this article previously published by Portland Press.

This article is being retracted from *Bioscience Reports* at the request of the Editor-in-Chief and the Editorial Board following receipt of a notification from a reader, alerting the Editorial Board to two sets of duplicated images in Figure 4D.

The authors have been contacted in regards to the retraction, and have not responded to the Journal's queries and the concerns raised. As correction of the article cannot be considered without input from the authors, the Editorial Board stand by the decision to retract the article.

